# Rapid metabolic control after primary islet transplantation: relevance for stem cell-derived islet product development

**DOI:** 10.3389/ti.2026.17176

**Published:** 2026-10-07

**Authors:** Juan R. Alvarez-Dominguez, Mai Liu, Carla Sawan, Scott A. Soleimanpour, Chengyang Liu, Ali Naji, Michael R. Rickels

**Affiliations:** 1 Perelman School of Medicine, University of Pennsylvania, Philadelphia, PA, United States; 2 Saint George University of Beirut, Beirut, Lebanon; 3 University of Michigan, Ann Arbor, MI, United States

**Keywords:** C-peptide, glucose metabolism, islet cells, stem cell derived beta cells, type 1 diabetes

Dear Editors,

Islet replacement therapy, including human primary and pluripotent stem cell-derived islet (SC-islet) transplants, is an effective treatment for individuals with type 1 diabetes experiencing severe hypoglycemia and glycemic liability. However, little is known about the timeline for achieving peri-transplant metabolic control. At the University of Pennsylvania, eleven individuals underwent allogeneic islet transplantation under the CIT07 protocol [1] between 2008 and 2012, with detailed long-term metabolic outcomes reported [2, 3]. Experience indicates that islets function immediately and contribute to reaching on-target glucose control within days post-transplant. Understanding these early metabolic effects may augment benchmarks to evaluate the speed and quality of diabetes reversal [2, 3] and inform schedules for assessing endpoints in future efforts to restore a functional islet β-cell mass using SC-islets. We previously reported on the functional islet β-cell mass from our CIT07 cohort of primary islet recipients at Day 75 post-transplant [2]. Here, we examine the peri-transplant metabolic control in all eleven recipients during these first 75 days.

Peri-transplant management of islet recipients involves intensive insulin therapy to provide “islet rest” during the critical period of revascularization of intraportally delivered islets. The aim is near-normoglycemia (fasting and pre-prandial glucose 80–120 mg/dL, post-prandial glucose <180 mg/dL) for at least several weeks. This allows recovery from instant blood-mediated inflammatory response (IBMIR), islet arterialization, and adequate oxygenation before imposing a high secretory demand on the engrafted β-cells [2]. Since primary islets isolated from deceased donors are developmentally and functionally mature, they continue to sense glucose and secrete insulin immediately post-transplant, despite the stress of isolation and the relatively hypoxic transplant environment before arterialization. For this reason, administering exogenous insulin to reduce the need for β-cell insulin secretion, along with avoiding hyperglycemia, is crucial to reduce metabolic demand on transplanted islets during the engraftment period. The inevitable glucose-dependent nutrient-triggered insulin secretion from transplanted islets nonetheless contributes to achieving target glucose control. To illustrate this, we present metabolic control outcomes during the first 75 days following an initial primary islet infusion for our previously described cohort [3] (Figure 1).

**FIGURE 1:**
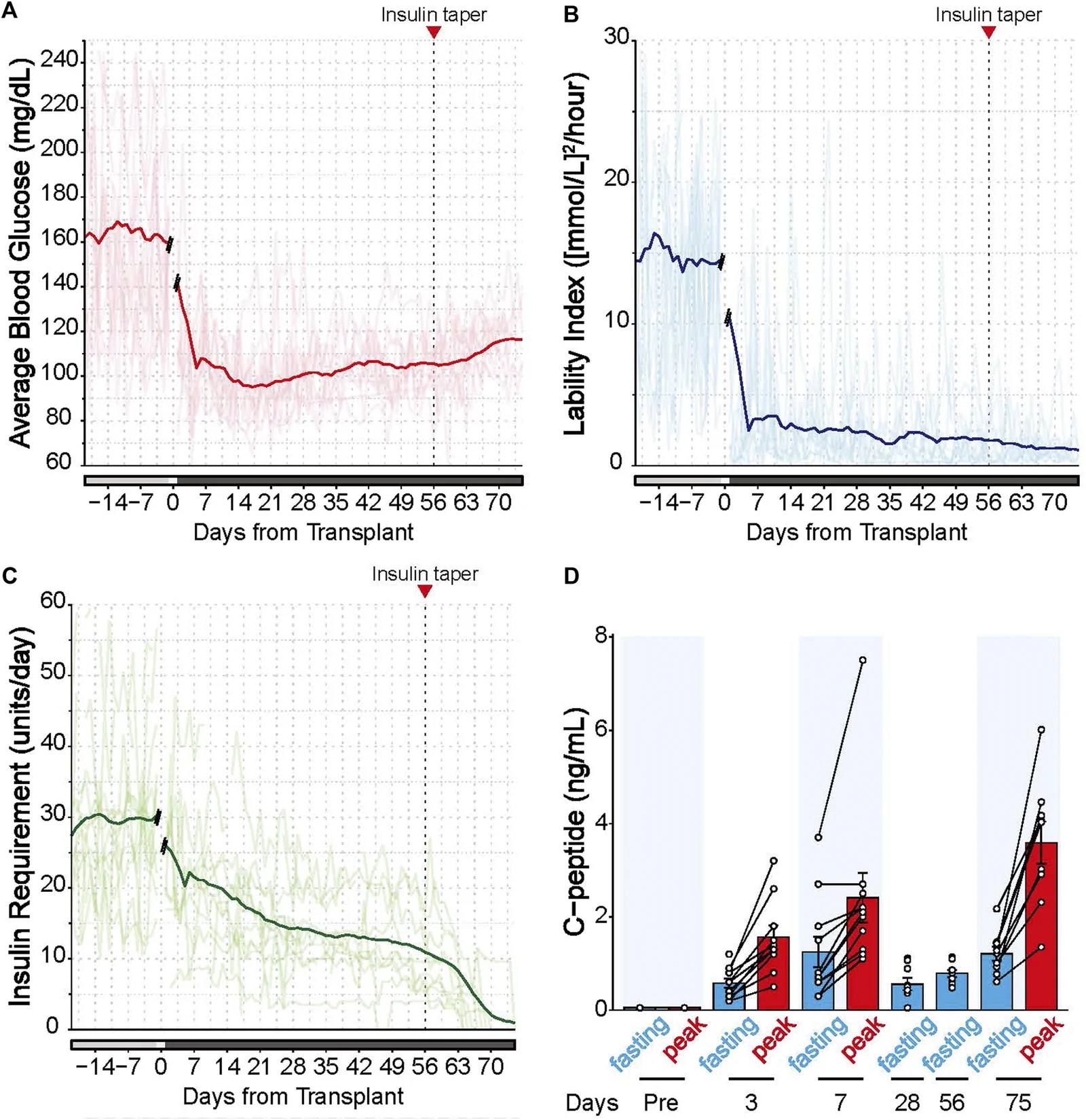
Metabolic control outcomes for the first 75 days following an initial primary islet infusion. Daily average blood glucose **(A)**, glycemic lability **(B)**, and total daily insulin requirement **(C)** are plotted relative to the day of primary islet transplantation (Day 0). Individual recipient data are plotted as thin lines, and the cohort-level running average as a bold line. C-peptide levels **(D)** were measured fasting and following meal stimulation before transplantation and at the indicated post-transplant days. The C-peptide lower limit of detection was 0.05 ng/mL. Bars plot mean ± SE data, with individual recipient data shown as dots. Pre-to post-transplant measures differed significantly for all metabolic outcomes [*P* < 0.01; paired two-tailed t-test **(A,C)**; Wilcoxon signed-rank test **(B)**].

The eleven individuals (four men, seven women) were median (range) age 49 years (28–63), with type 1 diabetes duration 34 years (12–50), BMI 24 kg/m^2^ (19–26), and insulin requirements 0.47 units/kg/day (0.24–0.78) at the time of receiving a median of 7,879 islet equivalents per kg body weight (5,277–12,073). The islets for each infusion were isolated from a single donor pancreas [4]. Induction immunosuppression was with anti-thymocyte globulin and etanercept, and maintenance immunosuppression with low-dose tacrolimus and sirolimus as previously described [1, 2]. Blood glucose was monitored >4 times daily, averaged daily and every 7 days to obtain a weekly average blood glucose. Weekly glycemic lability indexes were calculated from all blood glucose measurements recorded at least an hour apart, as previously described [5], to measure temporal glucose variability. Insulin requirements were totaled each day and averaged every 7 days to calculate a weekly insulin requirement. Endogenous insulin secretion was estimated from serum C-peptide levels measured fasting and following meal stimulation, as follows: pre-transplant and on Day 75 post-transplant, C-peptide was measured fasting and at 60- and 90-min following ingestion of 6 mL/kg (up to 360 mL) Boost® High Protein. On Days 3 and 7 post-transplant, C-peptide was measured fasting and at 60 and 120 min following a routine breakfast, with bolus insulin administered. On Days 28 and 56 post-transplant, C-peptide was measured fasting only.

Glucose control improved immediately after transplant, as indicated by pre-to post-transplant reductions in blood glucose (median 148 to 104 mg/dL; *P* < 0.01; Figure 1A) and glycemic lability (median 14.5 to 1.0 [mmol/L]^2^/hour; *P* < 0.01; Figure 1B) starting within the first week following islet infusion. This improved glucose homeostasis was accompanied by a reduction in insulin requirement (median 27 to 12 units/day; *P* < 0.001; Figure 1C), which declined weekly until achieving a plateau between weeks 4 and 8 at <50% of the pre-transplant insulin requirement. Between Days 56 and 75 post-transplant, all islet recipients tapered off exogenous insulin. While blood glucose increased modestly during this interval, all recipients met glucose targets with minimal glucose variability, completing a Day 75 mixed-meal tolerance test (MMTT) off exogenous insulin. Fasting and stimulated C-peptide were undetectable (<0.05 ng/mL) in all recipients prior to islet transplantation but were detectable at clinically meaningful concentrations at Day 3, progressively increasing through to the Day 75 MMTT (Figure 1D). At Day 75, all recipients had a fasting glucose <120 mg/dL; 7 of the 11 had a 90-min MMTT glucose <180 mg/dL, while 4 had a 90-min MMTT glucose ≥180 mg/dL that qualified them to receive a second islet infusion (data not shown). Of these 4 recipients, two had peak MMTT-stimulated C-peptide below, and two above, the 2.93 ng/mL threshold reported by the Collaborative Islet Transplant Registry for predicting insulin-independence [6]. All 7 recipients with 90-min MMTT glucose <180 mg/dL had MMTT-stimulated C-peptide above this threshold.

These data demonstrate the functional consequences of primary human islet transplants on metabolic control. Insulin therapy to provide “islet rest” avoids glucose toxicity while islet oxygenation and functionality improve, as evidenced by gradual increases in fasting and stimulated C-peptide levels. Despite the continued intensive insulin therapy, primary human islets function immediately after transplantation and contribute to the achievement of on-target glucose control for the recipient within the first days post-transplant. Full function of each primary human islet graft was previously demonstrated for this cohort by Day 75, with comparable β-cell secretory capacity by glucose-potentiated arginine testing between Day 75 and Day 365 [2]. Moreover, the MMTT-stimulated C-peptide levels at Day 75 are consistent with those identified through long-term follow-up in the Collaborative Islet Transplant Registry as predicting optimal outcomes for islet transplant alone recipients.

Unlike primary islets, SC-islets are molecularly and functionally immature [7, 8], and lack the precision, kinetics, and extent of insulin release seen in adult islets [9]. This delays the therapeutic benefit of SC-islet transplants, with >120 days needed on average for intraportal SC-islet transplant recipients to lower exogenous insulin needs by 50% [10], in contrast to ∼21 days for primary islet recipients (Figure 1C). The >5-fold time delay reflects the fact that SC-islets require a “maturation” period to gain increasing glucose-dependent insulin secretion beyond the engraftment period, as reflected by MMTT results [10]. The SC-islet transplantation study estimated cell dosing based on primary islet experience, following the same protocol for intraportal delivery and immunosuppression as in CIT07. Ten of 12 recipients of SC-islets eventually became insulin-independent with a single infusion, similar to the 7 of 11 in our CIT07 cohort. As achieving insulin independence supports the establishment of a reserve capacity for insulin secretion, these results suggest that delayed functional maturation, rather than insufficient engrafted SC-islet mass, explains the delay in glucose improvement.

Future developments in SC-islet engineering may wish to bridge this critical gap in functionality that delays assessment of adequacy of the delivered islet dose, engraftment, and immune evasion for each recipient. Primary graft non-function, IBMIR, and acute rejection can all be detected through metabolic function testing over the first days to weeks of primary islet transplantation. However, these mechanisms for graft failure may not be identified for SC-islets while awaiting *in vivo* functional maturation. Early metabolic control outcomes for primary islets may serve as useful functional benchmarks for a fully mature SC-islet product when assessing longer-term functional outcomes. Future advances in SC-islet development that enable early functional assessment may also help align expectations with evolving regulatory standards [11].

## Data Availability

The raw data supporting the conclusions of this article will be made available by the authors, without undue reservation.

## References

[1] Hering BJ Clarke WR Bridges ND Eggerman TL Alejandro R Bellin MD Phase 3 trial of transplantation of human islets in type 1 diabetes complicated by severe hypoglycemia. Diabetes Care (2016) 39:1230–40. 10.2337/dc15-1988 27208344 PMC5317236

[2] Rickels MR Liu C Shlansky-Goldberg RD Soleimanpour SA Vivek K Kamoun M Improvement in beta-cell secretory capacity after human islet transplantation according to the CIT07 protocol. Diabetes (2013) 62:2890–7. 10.2337/db12-1802 23630300 PMC3717864

[3] Flatt AJ Matus AM Gallop RJ Markmann E Dalton-Bakes C Peleckis AJ Beta-Cell secretory capacity predicts metabolic outcomes over 6 years following human islet transplantation. Diabetes (2025) 74:749–59. 10.2337/db24-0729 39630971 PMC12015140

[4] Ricordi C Goldstein JS Balamurugan AN Szot GL Kin T Liu C National institutes of health-sponsored clinical islet transplantation consortium phase 3 trial: manufacture of a complex cellular product at eight processing facilities. Diabetes (2016) 65:3418–28. 10.2337/db16-0234 27465220 PMC5079635

[5] Senior PA Bellin MD Alejandro R Yankey JW Clarke WR Qidwai JC Consistency of quantitative scores of hypoglycemia severity and glycemic lability and comparison with continuous glucose monitoring system measures in long-standing type 1 diabetes. Diabetes Technology and Ther (2015) 17:235–42. 10.1089/dia.2014.0289 25629445 PMC4447028

[6] Baidal DA Ballou CM Rickels MR Berney T Pattou F Payne EH Predictive value of C-Peptide measures for clinical outcomes of beta-cell replacement therapy in type 1 diabetes: report from the collaborative islet transplant registry (CITR). Diabetes Care (2023) 46:697–703. 10.2337/dc22-1155 36657975 PMC10148684

[7] Alvarez-Dominguez JR Donaghey J Rasouli N Kenty JHR Helman A Charlton J Circadian entrainment triggers maturation of human *in vitro* islets. Cell Stem Cell (2020) 26:108–22 e110. 10.1016/j.stem.2019.11.011 31839570

[8] Augsornworawat P Maxwell KG Velazco-Cruz L Millman JR . Single-cell transcriptome profiling reveals beta cell maturation in stem cell-derived islets after transplantation. Cell Rep (2020) 32:108067. 10.1016/j.celrep.2020.108067 32846125 PMC7491368

[9] Balboa D Barsby T Lithovius V Saarimaki-Vire J Omar-Hmeadi M Dyachok O Functional, metabolic and transcriptional maturation of human pancreatic islets derived from stem cells. Nat Biotechnol (2022) 40:1042–55. 10.1038/s41587-022-01219-z 35241836 PMC9287162

[10] Reichman TW Markmann JF Odorico J Witkowski P Fung JJ Wijkstrom M Stem cell-derived, fully differentiated islets for type 1 diabetes. N Engl J Med (2025) 393:858–68. 10.1056/NEJMoa2506549 40544428

[11] Piemonti L . The last mile in beta-cell replacement therapy for type 1 diabetes: time to grow up. Transpl Int (2025) 38:14565. 10.3389/ti.2025.14565 40236754 PMC11998595

